# Office Visitors

**Published:** 1906-12

**Authors:** 


### OFFICE VISITORS.

They walked into the office unannounced, a short thick set fellow and a comely young woman somewhat taller. "Howdy do—you're the Doctor? Well this is my wife, we both want some work done. I'm scary and nervous but she aint, she can stand anything. When our kid come—"

"Now shut your mouth, Jim, or talk about our business," ejaculated his wife.

"Well any how she is game, but my teeth are the worse. Size up the job Doc. and let me know the least you will do it for and if you come anywhere in reason I'll close with you and not go to another office. My name is Jim Fustman and I'm the "waffle-man" out here at the Carnival. We've been traveling around with the show and haven't stopped long enough in one place to have our teeth worked on." We fixed an agreeable price and the work progressed, they taking alternate sittings. The third appointment found them on hand. Jim unwrapped a package and produced a bottle of wine. "I just couldn't stand it any longer without something to drink, so I went into a bar down the street and threw with a fellow for this four dollar bottle of Sherry and won. I guess you and me and the old girl will about take it up."

"Now, Jim, you know I'll take but little of it," was her reply.

We began on it and it was really of such an excellent flavor that we were tempted to join Jim in several of his imbibings. We mentioned to him that on a certain occasion we had had opened a century old bottle of Madiera for our special benefit. By this time the Sherry had evidently gotten good hold of Jim's imagination and tongue.

"Well, now, Doc, I befriended a tramp once and, yes, he would cut wood. He was a tramp from bad luck and got stranded in our town. I gave him a breakfast, and do you know he sawed and cut up a cord of wood in our yard. Well, a few years after that as luck would have it, I was tramping the streets of Chicago with only ten dollars in my pocket, when a well dressed man stepped up to me and said, 'I know you. Come along with me.' I followed to his office in a bank. He was the fellow I had took care of. His luck had changed and he was rich. He made me tell him my condition and then he said he was going to pay me back for that breakfast I gave him. He took me to his tailor and gave me a suit of clothes, and then to his rooms—he was a bachelor—and made his valet give me a bath into which there was three quarts of Champagne emptied. Then when I got out we drank twenty-year-old whiskey and went to dinner. Gosh, how I eat! Wuss, I guess, than he did my slapjacks. Then to the theater, and next day an automobile ride and offered me a job in the bank."

We called time on Jim at this stage so as to finish up our work, and we were generous enough not to pry into the secret as to how he got down on luck again, and his wife kept her tongue. We finished, was paid, shook and separated.

